# Time-resolved fluorescence study of excitation energy transfer in the cyanobacterium *Anabaena* PCC 7120

**DOI:** 10.1007/s11120-020-00719-w

**Published:** 2020-02-19

**Authors:** Parveen Akhtar, Avratanu Biswas, Nia Petrova, Tomas Zakar, Ivo H. M. van Stokkum, Petar H. Lambrev

**Affiliations:** 1 Biological Research Centre, Szeged, Temesvári krt. 62, Szeged, 6726 Hungary; 2 ELI-ALPS, ELI-HU Nonprofit Ltd., Wolfgang Sandner u. 3, Szeged, 6728 Hungary; 3 Doctoral School of Biology, University of Szeged, Közép Fasor 52, Szeged, 6726 Hungary; 4 Department of Physics and Astronomy and LaserLaB, Faculty of Science, Vrije Universiteit Amsterdam, De Boelelaan 1081, 1081 HV Amsterdam, The Netherlands; 5 Institute of Biophysics and Biomedical Engineering, Bulgarian Academy of Sciences, Acad G. Bontchev Str., Bl. 21, 1113 Sofia, Bulgaria

**Keywords:** Allophycocyanin, Light harvesting, Photosynthesis, Photosystem I, Phycobilisomes, Phycocyanin

## Abstract

Excitation energy transfer (EET) and trapping in *Anabaena variabilis* (PCC 7120) intact cells, isolated phycobilisomes (PBS) and photosystem I (PSI) complexes have been studied by picosecond time-resolved fluorescence spectroscopy at room temperature. Global analysis of the time-resolved fluorescence kinetics revealed two lifetimes of spectral equilibration in the isolated PBS, 30–35 ps and 110–130 ps, assigned primarily to energy transfer within the rods and between the rods and the allophycocyanin core, respectively. An additional intrinsic kinetic component with a lifetime of 500–700 ps was found, representing non-radiative decay or energy transfer in the core. Isolated tetrameric PSI complexes exhibited biexponential fluorescence decay kinetics with lifetimes of about 10 ps and 40 ps, representing equilibration between the bulk antenna chlorophylls with low-energy “red” states and trapping of the equilibrated excitations, respectively. The cascade of EET in the PBS and in PSI could be resolved in intact filaments as well. Virtually all energy absorbed by the PBS was transferred to the photosystems on a timescale of 180–190 ps.

**Electronic supplementary material:**

The online version of this article (10.1007/s11120-020-00719-w) contains supplementary material, which is available to authorized users.

## Introduction

Light harvesting in cyanobacteria relies on phycobilisomes (PBS)—the large multimeric water-soluble assemblies of phycobiliproteins (PBPs) and linker proteins that efficiently absorb light in the spectral range between 550 and 650 nm (Harris et al. 2018; Bar-Eyal et al. 2018). PBS are commonly described as hemidiscoidal structures consisting of PBP trimers (αβ)_3_ and hexamers (αβ)_6_ assembled together with linker proteins into a central core of allophycocyanin (APC) with radiating rods containing phycocyanin (PC) and in some species phycoerythrin (PE) or phycoerythrocyanin (PEC) (Arteni et al. 2009; MacColl 2004). There are, however, large variations in the PBS organization and composition and deviations from this fundamental design depending on the species and growth conditions. Absorbed energy is transferred to the photosystems with a very high quantum efficiency (Scott et al. 2006; Glazer 1984)—PBS are generally considered as antenna for photosystem II (PSII) but they can also supply excitation energy to photosystem I (PSI), either indirectly via “spillover” from PSII or directly (Mullineaux 1994; Liu et al. 2013; Chukhutsina et al. 2015).

Excitation energy transfer (EET) has been studied in isolated PBPs (Holzwarth et al. 1990; Debreczeny et al. 1993; Switalski and Sauer 1984; Choubeh et al. 2018), PBS substructures (Sandström et al. 1988; Zhao et al. 1998), intact PBS (Tian et al. 2012; Nganou et al. 2016; Zhang et al. 1997; van Stokkum et al. 2018) as well as in whole cells of various species (Mullineaux and Holzwarth 1991; Tian et al. 2011; Acuña et al. 2018a, b; Chukhutsina et al. 2015), including *Anabaena variabilis* (Bittersmann et al. 1988; Nultsch et al. 1990). Despite the abundance of experimental data and models, there are contradicting views about the kinetics and rate-limiting steps of EET in the PBS and between them and the photosystems—partly because of the sheer complexity of the PBS. Individual PBP—PC or APC—contain phycobilin (PCB) pigments with different spectral properties and EET between them occurs on timescales from hundreds of femtoseconds to several tens of picoseconds in isolated (αβ)_3_ trimers (Choubeh et al. 2018; Debreczeny et al. 1993; Zhao et al. 1998; Holzwarth et al. 1990). Association of the trimers into hexamers accelerates the equilibration because of more parallel contacts between the pigments and EET along the PBS rods (Holzwarth 1991) can be faster than equilibration within the individual subunits (Suter and Holzwarth 1987). The overall equilibration between the rods and the APC core is found to be in the order of 20 to 120 ps, depending on the size and number of rods (Holzwarth 1991; Tian et al. 2012). EET between the PBS and the photosystems has been proposed to be either very slow (Mullineaux and Holzwarth 1991) or very fast (Tian et al. 2011) and equilibration within the assembled core is not well defined. An elaborated functional compartmental model of EET in *Synechocystis* PCC 6803 has been recently reported (van Stokkum et al. 2018), according to which the slowest EET rates are between the core cylinders (115–145 ps) and between the rods and the core (68–115 ps). By time-resolved fluorescence, Acuña et al. (2018b) reported a 20 ps EET time from the PBS terminal emitter to PSII, i.e. faster than the EET within the PBS.

The PBS of the nitrogen-fixating cyanobacterium *Anabaena* PCC 7120 (hereafter called *Anabaena*) belong to the same hemidiscoidal family, as for instance the well-studied *Synechocystis* 6803, but are different in several ways (Ducret et al. 1998; Glauser et al. 1992). They contain up to eight rods (compared to six in *Synechocystis*), composed of not only PC but also PEC hexamers absorbing with a maximum at 575 nm owing to the bound phycoviolobilin chromophore (PVB). The penta-cylindrical APC core contains two supplementary flanking cylinders in addition to the two basal and the top cylinder found in the tri-cylindrical core of *Synechocystis*. *Anabaena* has unusual tetrameric organization of PSI (Watanabe et al. 2014), whose structure has been recently revealed by cryoelectron microscopy (Kato et al. 2019). The so-called “red” chlorophylls (Chls) in PSI absorbing light at longer wavelengths than the reaction centre (RC) (Gobets et al. 2001; El-Mohsnawy et al. 2010; Karapetyan et al. 2006) are especially prominent in *Anabaena*, which shifts the fluorescence emission maximum by 8 nm compared to *Synechocystis*. Supercomplexes containing tetrameric PSI and a special type of PBS containing only rods of PEC and PC and the rod-core linker CpcL have been observed in vegetative and nitrogen-fixating *Anabaena* cells (Watanabe et al. 2014). The CpcL-PBS is thought to be a specific antenna for PSI (Kondo et al. 2007; Liu et al. 2019; Niedzwiedzki et al. 2019); however, it is not yet studied in detail. The specific characteristics of the PBS and PSI in *Anabaena* are expected to affect EET in the complexes and consequently in the cells.

In this work, we applied steady-state and time-resolved fluorescence spectroscopy to study the dynamics of EET in isolated PBS, PSI and intact filaments of *Anabaena* PCC 7120. We show that spectral equilibration in the PBS occurs on timescales up to 125 ps and is a rate-limiting factor in the kinetics of intact cells where most excitations on the PBS are trapped on a timescale of about 180 ps, i.e. slower than the charge separation in either PSI or PSII. We further resolved the kinetics of exciton equilibration and trapping in tetrameric PSI both in vivo and in the isolated complex.

## Materials and methods

### Sample preparation

#### Cell growth conditions

Vegetative cells of *Anabaena variabilis* (PCC 7120) were cultivated in BG-11 (Rippka et al. 1979) medium supplemented with 5 mM HEPES–NaOH (pH 7.5) at 30 °C under continuous white light illumination at an intensity of 40 μmol photons m^−2^ s^−1^. Cultures were aerated on a gyratory shaker operating at 100 rpm.

#### Isolation of PBS

PBS were prepared from *Anabaena* filaments according to Garnier et al. (1994) with some adjustments. Briefly, photoautotrophically grown cells were centrifuged to pellet at 7000×*g* at 25 °C. The pellet was resuspended in 0.75 M phosphate buffer, 1 mM benzamidine hydrochloride hydrate, 1 mM ethylenediaminetetraacetic acid (pH 7.0), and 1 mM of phenylmethylsulfonyl fluoride and homogenized by using a bead-beater homogenizer followed by centrifugation at 3000×*g* for 5 min at 14 °C to remove the filament debris. The supernatant was then treated with 3% Triton-X100 with continuous stirring for 45 min at room temperature in dark and centrifuged at 70,000×*g* for 30 min to remove the unsolubilized material. The appropriate sample fraction was collected and loaded onto a sucrose density step gradient (1 M, 0.75 M, 0.5 M, 0.25 M sucrose) and centrifuged for 16 h at 26,000 rpm, 14 °C for further purification. The gradient fraction containing PBS was then collected and characterized by steady-state spectroscopy for further use.

#### Isolation of photosystem I

PSI was isolated from the 1-week-old cells according to Vajravel et al. (2017). Briefly, thylakoid membranes were isolated in a medium containing 25% glycerol, 0.5 mM phenylmethanesulfonyl fluoride and 1 mM benzamidine by breaking the cells by glass beads in a beater. Isolated thylakoid membranes were further solubilized with 2% *n*-dodecyl β-d-maltoside (β-DDM) and loaded on a stepwise (6 steps, 0.2–0.9 M) sucrose gradient containing 20 mM HEPES (pH 7) and 0.05% of β-DDM followed by centrifugation at 220,000×*g* for 14 h at 4 °C. PSI-containing fractions of the gradient were collected by a syringe. The sample was washed in a medium containing 0.03% β-DDM and concentrated using Amicon Ultra filters (Millipore), characterized and frozen in liquid N_2_ and stored at − 80 °C until use. The purity of the samples was confirmed by SDS-PAGE (Supplementary Fig. S1).

### Absorption and fluorescence spectroscopy

Absorption spectra were recorded between 350 and 750 nm at room temperature using an Evolution 500 dual-beam spectrophotometer (Thermo Scientific). Samples were diluted with their corresponding media to an absorbance of one at the red maximum. Measurements were performed in a standard glass cell of 1 cm optical path length.

Fluorescence emission spectra at 77 K were recorded with a Fluorolog 3 double-monochromator spectrofluorometer (Horiba Jobin–Yvon, USA). In the case of isolated samples, 40–50 μl of solution with absorbance of 0.3 was evenly placed onto a Whatman GF/C glass microfiber filter and immersed in liquid nitrogen in a Dewar glass vessel. For measuring fluorescence from intact cells, a culture volume containing 5 µg Chl was filtered onto a Whatman GF/C filter. Emission spectra were recorded in the range of 620–800 nm with excitation at 460 or 580 nm and spectral bandwidth of 5 and 2 nm for excitation and emission, respectively.

### Time-resolved fluorescence

Time-correlated single-photon counting (TCSPC) measurements at room temperature were done using a FluoTime 200/PicoHarp 300 spectrometer (PicoQuant, Germany) equipped with a microchannel plate detector (R3809U, Hamamatsu, Japan) as described in Akhtar et al. (2016). Excitation pulses at 460 or 580 nm at 20 MHz repetition rate, ~ 0.1 pJ energy, were obtained from a Fianium WhiteLase Micro (NKT Photonics, UK) supercontinuum laser coupled to a monochromator. The total instrument response (IRF) width was 48–50 ps, measured using 1% Ludox as scattering solution. Isolated PBS and PSI complexes were diluted to an absorbance of 0.03 at the excitation wavelength and intact filaments to 0.05 at 750 nm and circulated through a 1.5-mm pathlength flow cell. The fluorescence decays were recorded at emission wavelengths between 600 and 800 nm with 6 or 8 nm step to construct time-resolved emission spectra. Global multiexponential lifetime analysis with IRF reconvolution was performed using MATLAB.

To keep the PSII RC from being closed by the excitation beam, the cell suspension was circulated at a flow rate of 4 ml/min. Alternatively, measurements with closed PSII RCs were performed by adding 20 µM DCMU to the medium, additional background light and lowering the flow rate ~ tenfold.

## Results

### Absorption and fluorescence spectra

**Fig. 1:**
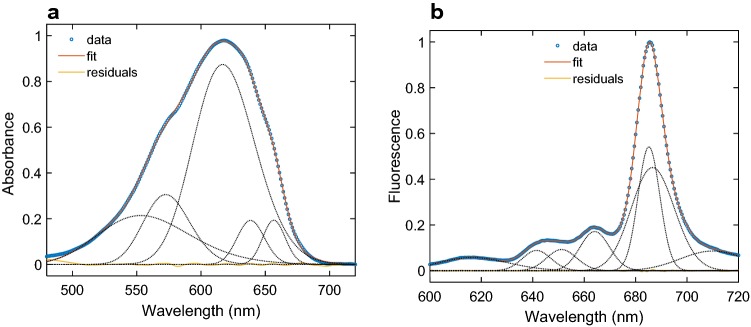
Steady-state absorption and fluorescence spectra of isolated PBS from *Anabaena* PCC 7120 and Gaussian decomposition analysis. **a** Absorption spectrum at room temperature and best-fit Gaussian components centred at 553, 573, 617, 639, 657 nm. **b** Fluorescence emission spectrum recorded at 77 K (excitation 580 nm) and best-fit Gaussian components centred at 617, 641, 651, 664, 685, 686, 710 nm

PBS and PSI complexes isolated from *Anabaena* were characterized by steady-state absorption and fluorescence spectroscopy (Fig. 1). The absorption spectrum of PBS is dominated by a broad band with a maximum at 620 nm, corresponding to absorption of PC and shoulders on the blue and red side of the maximum. Gaussian decomposition analysis revealed additional bands at 573 nm, originating from PEC, 638 nm—from red-shifted PC, and 656 nm, corresponding to APC. Overall, the absorption spectrum is very similar to those previously published for *Anabaena* (Jallet et al. 2014). The fluorescence emission spectrum (Fig. 1b) at 77 K has a maximum at 685 nm reflecting the terminal emitter APC pigments. Additionally, several bands with relatively small amplitudes are observed on the blue side and a broad vibronic tail extends to the near-infrared region. The spectrum is also broadly similar to the earlier reported, only slightly more structured (Jallet et al. 2014), allowing us to verify the peak positions by Gaussian decomposition. The analysis shows the presence of two spectral forms of APC, emitting at 664 and 685 nm, PEC emitting around 617 nm and two forms of PC at 640 and 650 nm. It must be noted though that the Gaussian fit components of both the absorption and emission spectra neither represent all spectral forms in the complex nor their actual bandwidths or stoichiometric ratios. For example, the broader absorption band at 553 nm and emission band at 685 nm likely cover multiple spectral forms. The longest-wavelength emission component may be due to vibrational overtones. Because excitation energy is efficiently transferred to the terminal emitters (685 nm), all other bands have relatively low intensity (< 20% of the main maximum), despite the high pigment abundance. The emission maximum of isolated PBS at room temperature is observed around 670 nm and the spectrum is broader and less featured than at 77 K (Supplementary Fig. S2a).

**Fig. 2:**
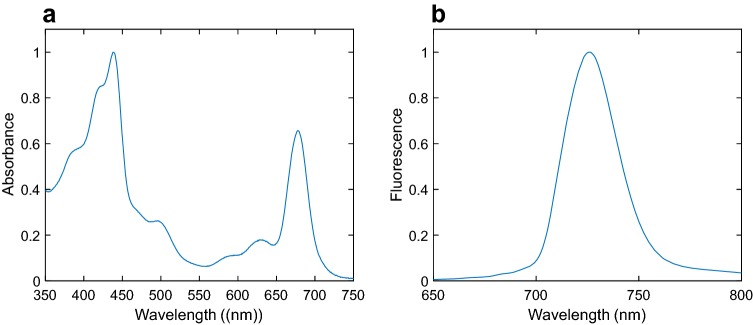
Absorption and fluorescence spectra of isolated tetrameric PSI form *Anabaena.***a** Absorption spectrum at room temperature. **b** Fluorescence emission spectrum recorded at 77 K (excitation 460 nm)

The absorption and fluorescence spectra of PSI isolated by sucrose gradient ultracentrifugation are shown in Fig. 2. The absorption spectrum is generally similar to the spectrum of PSI isolated from other cyanobacteria (Andrizhiyevskaya et al. 2002; Lundell et al. 1985; Rögner et al. 1990). The main absorption bands correspond to Chl *a* Soret and *Q* transitions and the peak at 500 nm originates from β-carotene. The absorption spectrum extends well beyond 700 nm owing to the presence of low-energy “red” Chls, the number of which varies among different cyanobacterial species (Gobets et al. 2001; Karapetyan et al. 2006). The 77 K fluorescence emission spectrum (Fig. 2b) is very similar to the recently reported spectrum of PSI tetramers from *Anabaena* (Kato et al. 2019). As excitation energy is trapped by the red Chls at low temperature, the emission maximum is at 725 nm and the intensity at wavelengths shorter than 700 nm is negligible—in contrast, the fluorescence maximum at room temperature is at 684 nm (Supplementary Fig. S2b).

**Fig. 3:**
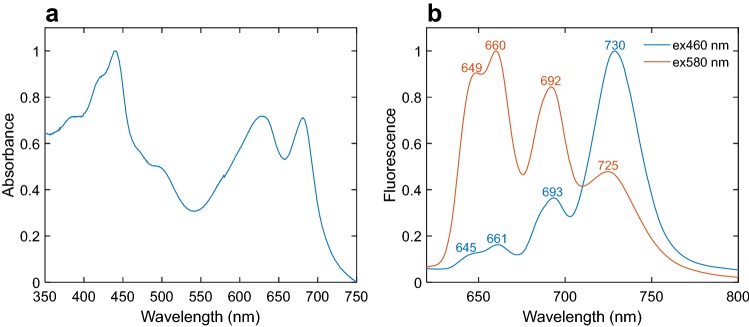
Absorption and fluorescence spectra of intact cells. **a** Absorption spectrum at room temperature. **b** Fluorescence emission spectra recorded at 77 K

The absorption spectrum of intact *Anabaena* cells and the fluorescence emission spectrum recorded at 77 K (Fig. 3) are similar to those previously published for the same species (Ogawa et al. 1970; Peterson et al. 1981). Between the Soret and *Q*_y_ absorption bands of Chl *a*, at 440 and 680 nm, respectively, the PBS absorption peaking at 628 nm is visible (Fig. 3a). For fluorescence emission spectra either 590 nm light was used, exciting mainly PC, or 460 nm light, exciting mainly Chls in PSI and PSII. The 77 K fluorescence spectrum excited at 460 nm has a maximum at 730 nm showing that most of the emission originates from the red Chls in PSI (Fig. 3b). The peak around 693 nm that can be attributed to PSII has much lower intensity, because of the relative abundance of PSI Chls. The emission spectrum with 580 nm excitation has well-defined peaks at 649, 660, 692 and 725 nm. The emission peaks at 649 and 660 nm correspond to PC and APC, 692 nm to PSII and 725 nm to PSI emission. The areas under the PSI and PSII peaks are comparable, indicating that PBS transfers to both PSI and PSII with similar efficiency, assuming that the fluorescence quantum yield is similar for both bands.

Qualitatively similar dependence of the emission spectra on excitation wavelength is observed at room temperature; however, the spectra are generally blue-shifted and less featured (Supplementary Fig. S3). The strong excitation dependence of the spectra shows that fluorescence emission occurs before equilibration of the excitation energy between the PBS and the photosystems. The emission maximum upon 580 nm excitation is at 654 nm, whereas the spectrum recorded with 460 nm excitation has a maximum at 684 nm and a broad PSI shoulder around 710 nm.

### Time-resolved fluorescence spectroscopy

**Fig. 4:**
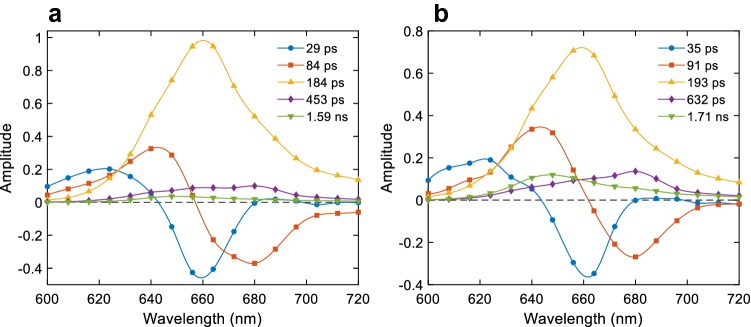
DAES obtained from five-component global lifetime analysis of the fluorescence decays of intact *Anabaena* PCC 7120 filaments upon excitation at 580 nm. **a** With open PSII RCs, **b** with closed PSII RCs

We applied time-resolved fluorescence spectroscopy to resolve the dynamics of EET within the PBS, the photosystems and between them in intact *Anabaena* filaments. Measurements were performed with two excitation wavelengths, 580 or 460 nm, predominantly exciting either PBS or Chls, respectively, and under conditions favouring open or closed PSII RCs. Representative DAES resulting from global lifetime analysis of the fluorescence recorded with 580 nm excitation are shown in Fig. 4. Minimum five exponential decay components were necessary for a good fit of the data (Supplementary Fig. S4). The resulting DAES have distinct shapes as they reflect emission from different groups of pigments (see the normalized DAES, Supplementary Fig. S5). The shortest resolved lifetimes are 28 ± 4 ps and 88 ± 5 ps (mean values ± standard deviation from nine independent experiments). The first lifetime is characterized by fluorescence decay at 620–630 nm and rise at 660 nm indicated by the positive and negative peaks in the DAES, respectively (Fig. 4a). The second lifetime shows decay at 640 nm and rise at 670–680 nm. The DAES strongly suggest EET from pigment pools emitting at the shorter wavelengths to acceptors emitting at the longer wavelengths. Therefore, we can conclude that the two components reflect EET between PC and APC pigments in the PBS. A decay component with a lifetime of 180 ± 10 ps dominates the kinetics in the 650–700 nm region; its maximum at 660 nm strongly suggests that it represents decay of excitations in APC. The 450 ± 20 ps decay lifetime, which has a relatively smaller contribution to the overall decay, can be ascribed to both PBS and PSII as it has peaks around 650–660 nm and 680 nm (Supplementary Fig. S5). The spectrum was found to vary somewhat between sample batches. Finally, a long-lived component (1.6 ± 0.1 ns) with emission maximum at 648 nm indicates a small (3%) fraction of energetically uncoupled PC.

Closing the PSII RCs in the presence of DCMU and additional background illumination brought about changes primarily in the three long-lived decay components (Fig. 4b). The 193 ps DAES consistently showed less emission at 680 nm (compare with the 184 ps DAES in open RCs). The fourth lifetime increased from 450 ± 20  to 590 ± 20 ps. Moreover, the relative amplitudes of the two longest-lived components increased significantly (see also Supplementary Fig. S6). Judging from the shape of the DAES, these components appear to originate from PSII as well as from the PBS.

**Fig. 5:**
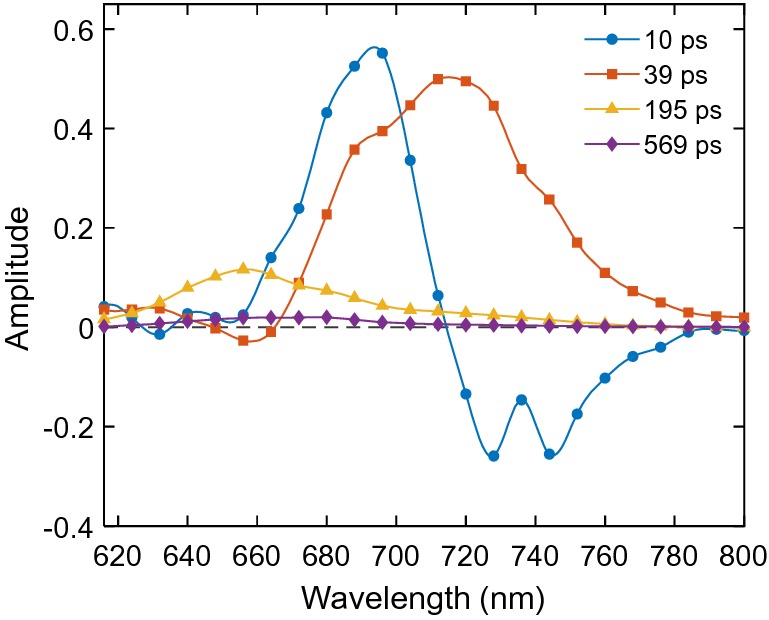
DAES obtained from global lifetime analysis of the fluorescence decays of intact *Anabaena* PCC 7120 filaments upon excitation at 460 nm

The fluorescence kinetics upon 460 nm excitation is markedly different, largely because the initial excitations reside on the photosystems rather than the PBS antenna. Two lifetimes dominate the kinetics above 670 nm—10 and 39 ps. The 10 ps lifetime was fixed—a marginally better numerical fit could be obtained with a value of 14 ps but with less realistic DAES (not shown). The first DAES (Fig. 5) shows fluorescence decay at 680 nm and rise at 720–740 nm and the second DAES shows overall decay with a maximum at 720 nm. Based on the lifetimes and DAES, these components can be assigned to energy equilibration and trapping in PSI. The long-lived components (195 ± 20 ps, 570 ± 50 ps, 1.7 ns) have lifetimes and DAES similar to their respective counterparts resolved upon 580 nm excitation but are of smaller relative amplitudes. A closer comparison of the long-lived DAES also reveals that all three components have higher relative amplitudes at 680 nm upon selective excitation of Chls—this was noticeable with either open or closed PSII RCs (Supplementary Fig. S6).

The excitation wavelength dependence of the fluorescence emission is also apparent in the stationary spectra, reconstructed from the DAES in “*F*_o_” or “*F*_m_” (i.e. preferentially open or closed PSII RCs, respectively), and in the calculated variable fluorescence spectra, *F*_v_  = *F*_m_ − *F*_o_ (Supplementary Fig. S7). Upon Chl excitation, the *F*_v_ spectrum peaks at 680 nm but lacks the distinct PSI-associated far-red emission band. Upon PBS excitation, there is a pronounced peak at 650 nm, which is mainly due to the increased amplitude of the nanosecond decay component under “*F*_m_” conditions. The *F*_v_/*F*_m_ values of 0.3–0.4, calculated from the time-resolved fluorescence, are lower than the typically observed values of 0.4–0.6 in dark-adapted cyanobacterial cultures (Campbell et al. 1998), possibly due to actinic effects of the measuring light and incomplete closure of the RCs.

**Fig. 6:**
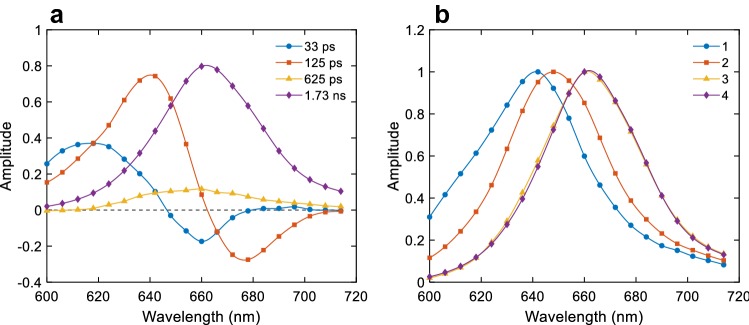
Global lifetime analysis of the time-resolved fluorescence kinetics of isolated PBS with 580 nm excitation. **a** Decay-associated emission spectra, **b** evolution-associated emission spectra (normalized to the maximum)

To further disentangle the kinetics of EET in the photosynthetic complexes, we performed separate measurements on isolated PBS and isolated PSI. Four components were required for a satisfactory fit of the fluorescence of isolated PBS in the wavelength range 600–720 nm after 580 nm excitation (Fig. 6). The first two lifetimes (33 ps and 125 ps) have DAES closely reminiscent of the 29 ps and 84 ps DAES resolved in intact filaments and representing EET within the PC rods and between the rods and the APC cores. The two longer lifetimes, 625 ps and 1.7 ns have near-identical DAES shape with maxima at 645 and 660 nm. The latter is the dominant decay component, whereas the faster component has a small amplitude. As a first approximation, the emission spectra of the main pigment pools can be obtained from a sequential irreversible kinetic model (Fig. 6b). The evolution-associated emission spectra (EAES) could be interpreted as three pigment pools: the first one emitting at 635–640 nm (PC) with a shoulder at 600–620 (PEC), the second emitting at 640–645 nm (PC), and the last at 660 nm with a shoulder at 680 nm (APC). Because the actual kinetic system is more complex, as discussed below, this is only a guiding assignment.

**Fig. 7:**
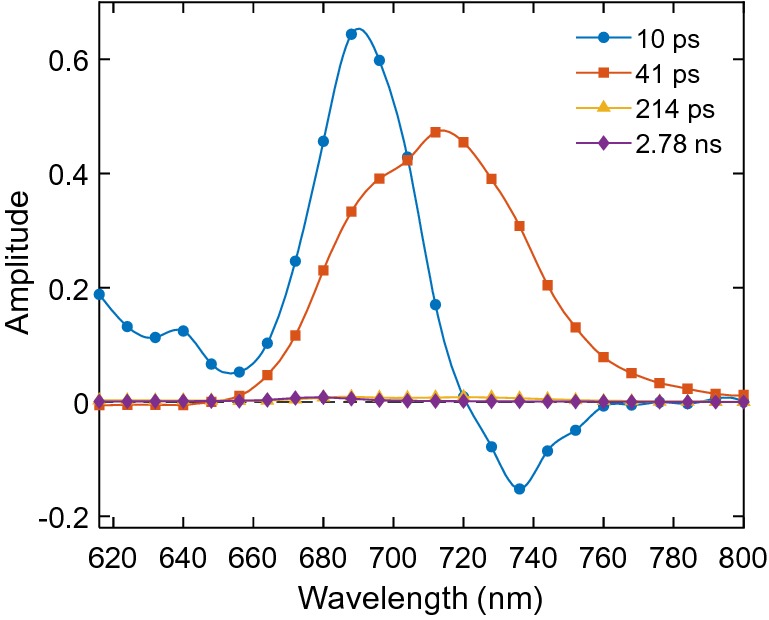
DAES obtained from global analysis of the fluorescence kinetics of isolated PSI from *Anabaena* PCC 7120 recorded with excitation at 460 nm

The fluorescence decay of PSI was followed by excitation at 460 nm. DAES obtained from four-exponential global analysis are shown in Fig. 7. In close similarity to the fluorescence of intact cells excited at 460 nm, two components with lifetimes of 10 ps and 41 ps dominate the kinetics. The 10 ps DAES has a positive peak at 685 nm and a negative peak around 730 nm, clearly representing energy equilibration between bulk PSI antenna Chls and low-energy “red” Chl forms. The 41 ps DAES has only positive amplitude with a peak at 720 nm and a shoulder at 688 nm, reflecting the trapping of equilibrated excitations in PSI (Fig. 7). Equilibration between the bulk and red forms is also observed in the raw time-resolved emission spectra as a shift of the maximum from 688 to 720 nm after 40 ps (Supplementary Fig. S8). Two long-lived components were required to fit the kinetics in a 4 ns window but their amplitudes are less than 1% of the main decay component. We hypothesize that these components originate from uncoupled pigments present in the sample; however, conclusive assignment cannot be made without detailed kinetic modelling analysis.

## Discussion

Understanding the mechanisms and pathways of energy transfer in the cyanobacterial cells is far from trivial, given the large size of the PBS antenna, the variety of assemblies and structures that the PBP can accommodate, and the possibilities for vertical and lateral energy transfer between different PBPs in the same PBS or between PBS in densely packed arrays (Liu et al. 2008; Eyal et al. 2017; Harris et al. 2018). This complexity would account for an enormous number of kinetic parameters in modelling the excitation dynamics (Saer and Blankenship 2017), which are difficult or impossible to measure directly; hence, a complete energy transfer model of the entire system is lacking. The PBP composition and architecture of the PBS varies among species, which has impact on the dynamics and efficiency of light harvesting. With the present work we aimed to gain further insight into the dynamics of excitation energy transfer and trapping in *Anabaena* PCC 7120 by comparing the fluorescence kinetics in intact cells and in the isolated major pigment–protein complexes, PBS and PSI. Taken separately, the experimental results confirm a number of observations well documented in the literature and present some new findings as discussed below. As the fluorescence kinetics in vivo and in vitro were obtained under the same measurement conditions and from the same cell cultures, the combined results form an experimental basis that could be used for testing and comparing different kinetic models.

### Energy transfer in isolated PBS

Global lifetime analysis of the fluorescence kinetics of isolated PBS resolved two decay lifetimes, 33 and 125 ps, that are clearly associated with energy transfer between PEC/PC in the rods, APC, and the terminal emitters, APC-E and APC-D, of the PBS core. Based on the maxima of the EAES, it could be concluded that the 33 ps lifetime represents EET within the rods—from PVB in PEC and the β-155 PCB chromophores in PC to the lower-energy β-84 and α-84 chromophores emitting at 640–650 nm (Debreczeny et al. 1993). Similarly, the 125 ps could be assigned to decay of PC and corresponding rise of emission from APC, i.e. to EET from the rods to the core. It is obvious from the DAES in Fig. 6 that the rise of the emission at wavelengths longer than 680 nm is described solely by the 125 ps component, which is evidently the overall energy transfer time to the terminal emitters in the APC core. The shape of the final DAES indicates decay of equilibrated excitations among the APC emitting around 660 nm and the terminal emitters found in the basal core cylinders. Since we do not resolve the equilibration term, we can conclude that it is faster than the experimental time resolution—which is in agreement with earlier models, e.g. recent kinetic models of energy transfer in *Synechocystis* assigns APC660–APC680 “intradisk” equilibration time of about 3 ps (van Stokkum et al. 2018; Acuña et al. 2018a).

The decay lifetimes and their amplitudes at different wavelengths (DAES) are in good agreement with earlier reports on the EET of isolated PBS from various cyanobacteria. Suter et al. (1984) measured the fluorescence decay of PBS from *Synechococcus *sp*.* upon 580 nm excitation and found that the fluorescence at 590 nm decays primarily with a 25 ps lifetime, attributed to EET from the sensitizing to the fluorescing chromophores in PC. They also found that the APC emission at 680 nm rises over 130 ps, representing the effective time constant for rod–core transfer.

It should be noted that the effective time constants of EET represent a sum of a large number of microscopic rate constants and generally cannot be attributed to any individual hopping step between specific pigment groups. This is the reason why the effective rod–core transfer time depends on the length of the rods and can be as short as 17–18 ps in particles containing only one PC trimer (Sandström et al. 1988; Zhang et al. 1997). Thus, the observed results (DAES) can be interpreted differently depending on the choice of kinetic model. The DAES of *Anabaena* PBS shown here closely resemble the simulated DAES by Suter & Holzwarth’s (1987) kinetic model for the rod–core assembly except for the lack of a 10 ps EET lifetime. According to their model, the excitation migration along the rods is fast and the overall kinetics is limited by transfer to the APC core, which was considered as a single “trap” component. Tian et al. (2012) recorded the fluorescence kinetics of PBS from *Synechocystis *sp. using a streak camera and reported DAES that were also remarkably similar to the ones presented here, except for an additional rise lifetime of 8 ps. Although we have not been able to resolve such a component in our TCSPC experiments, it is conceivable that the true rise term might be even shorter than 8 ps and beyond the experimental time resolution. In contrast to the earlier model by Suter and Holzwarth (1987), the 120 ps lifetime component was ascribed to EET within the APC core and equilibration between APC660 and the terminal emitters. This would seem a reasonable interpretation of the present results, considering that the 125 ps DAES has a negative maximum at 680 nm (Fig. 6). However, if we adopt a sequential kinetic model, the EAES show that the species being populated on a timescale of 33 ps emits with a maximum at 650 nm (PC) and the 125 ps lifetime populates a component with an emission maximum at 660 nm (APC). It can be concluded then that the former lifetime is associated with equilibration in the rods where the energy donors are PBV and blue-shifted (sensitizing) forms of PCB (e.g. the β155 chromophores) and the acceptors are chromophores emitting around 650 nm, presumably belonging to PC. This would fit the observed two spectral components in the 77 K emission spectra—at 640 and 650 nm. Therefore, it appears more likely that the rate-limiting step in the kinetics is between the rods and the core, rather than spectral equilibration within the APC core. This does not mean, however, that EET in APC has no influence on the overall dynamics. All APC trimers contain blue-shifted and red-shifted PCB forms, absorbing around 610 and 650 nm, respectively, with EET between them taking place in less than 1 ps to tens of ps (Holzwarth et al. 1990; Choubeh et al. 2018). Equilibration in APC trimers containing forms emitting at 680 nm (as in the basal ApcE and ApcF) has been shown to occur in 17 to 66 ps (Holzwarth et al. 1990; Zhao et al. 1998; Nganou et al. 2016). The effective lifetimes should be different in the fully assembled core. Therefore, both the 33 ps and the 125 ps lifetimes resolved in the intact *Anabaena* PBS probably involve pigments in PEC/PC as well as APC.

The additional decay lifetime in the range of 600–700 ps is uncovered here likely thanks to a higher signal-to-noise ratio in the TCSPC data. As the spectra of this and the main decay component at 1.7 ns are very similar, they originate from the same pigment groups. It is possible that the 600 ps decay reflects heterogeneity in the sample, like a subpopulation of mildly quenched APC. This quenching, however, is probably different from the photoprotective quenching of APC fluorescence induced by the orange carotenoid protein (OCP) (Kirilovsky and Kerfeld 2016), wherein a much higher deactivation rate is expected (Tian et al. 2011). The heterogeneity could thus be an artefact of the PBS isolation. However, it seems that it is an always present kinetic component, having approximately equal lifetime and relative amplitude in all tested preparations, and it is plausible that the same decay channel exists in vivo (see below). Alternatively, the decay lifetime may stem from excitation migration between subunits of the APC core, i.e. it is an intrinsic kinetic component. One could assume this to be specific feature of *Anabaena* due to the distinct architecture of the APC core. However, we could also resolve a similar 600–800 ps decay component in tri-cylindrical-core PBS from *Synechocystis *sp. (data not shown). The reason why it has not been reported earlier is most likely its relatively small amplitude.

### Photosystem I

The fluorescence of isolated PSI in the picoseconds time range exhibits essentially biexponential fluorescence decay kinetics, which is typical for cyanobacteria (Gobets and van Grondelle 2001). Byrdin et al. (2000) reported fluorescence lifetimes of 13 and 37 ps in PSI from *T. elongatus* with DAES very similar to the ones in Fig. 7 with one notable difference being the non-conservative shape of the 10 ps DAES. The positive and negative peaks in the DAES point to equilibration between the bulk antenna Chls in PSI emitting around 680 nm and the “red” Chls emitting at 710–720 nm. In Byrdin’s (2000) experiments, the rise amplitude at 720 nm was significantly larger than the corresponding decay amplitude at 680 nm, which was interpreted in terms of a kinetic model where excitations on the two pigment pools are trapped with different rate constants, assuming that trapping occurs before complete equilibration. Our results indicate that besides equilibration of bulk and red Chls there is already some trapping on the 10 ps time scale (cf. Figs. 5 and 7). This will be verified by target analysis in a subsequent study. The 41 ps DAES shows the trapping of equilibrated excitations in the RC. This two-component kinetics of PSI is well resolved also in vivo upon 460 nm excitation—the two decay components of 10 and 39 ps resolved in intact cells have spectra closely reminiscent of the corresponding DAES of isolated PSI.

As with other studies on cyanobacteria, we find that the great majority of Chls is connected with PSI and only a small fraction belongs to PSII. This is evident from the DAES of cells upon 460 nm excitation, which are dominated by the PSI-associated components (Fig. 5) as well as from the steady-state emission spectra recorded at 77 K (Fig. 3b).

### Energy transfer in intact cells

The time-resolved fluorescence kinetics of intact *Anabaena* filaments share a lot of similarities with the reported kinetics from cells of different cyanobacterial strains (Mullineaux and Holzwarth 1991; Tian et al. 2011; Acuña et al. 2018b). Upon direct excitation of the PBS rods, the fluorescence kinetics in a few hundred picoseconds reflects primarily EET within the PBS, which is apparent by the similar DAES observed in cells and isolated PBS. The principal difference is that coupling of the PBS to photosystems in intact filaments shortens the APC decay lifetime to 170–200 ps as opposed to 1.5–1.7 ns in the isolated PBS. By global analysis of the fluorescence kinetics of WT *Synechocystis* cells, Tian et al (2011) observed three rise terms—at 645, 660 and 680 nm, with lifetimes of 8, 39 and 122 ps, respectively. They assigned the lifetimes to EET within the PC rods (8 ps), between the rods and the core (39 ps), and from APC660 to the core terminal emitters and Chls (122 ps). The latter two components bear obvious similarity with the 29 and 84 ps DAES of *Anabaena* filaments (Fig. 4) but also to the 33 and 125 ps DAES in isolated PBS (Fig. 6). As was described above, a simple sequential kinetic scheme of these data would suggest a different assignment of the intermediate species, i.e. rise of PC emitting at 650 nm on a 30 ps timescale followed by transfer to APC (84–125 ps). This would fit with the PBS kinetics proposed by Holzwarth (Mullineaux and Holzwarth 1991; Suter and Holzwarth 1987) with rod–core equilibration occurring on 90–120 ps timescale in *Synechococcus* 6301. Interestingly, the PBS-associated lifetimes are consistently shorter in intact filaments than in isolated PBS. There could be several possible reasons for the observed differences. The decay lifetimes may be affected by EET between densely packed PBS (Harris et al. 2018) or between PBS and the photosystems. We cannot rule out the possibility of non-radiatively decaying PC in vitro, which would explain the non-conservative shape of the DAES in this case (Fig. 6a).

Tian’s results showed fluorescence decay at 660–680 nm with a lifetime of 195 ps (again closely comparable to the 184 ps lifetime we find in *Anabaena* filaments), assigned to excitation trapping via charge separation in the PSII RCs. A more detailed kinetic model describing the same data included EET from PC to APC660 with an effective time constant of about 50 ps and from APC660 to APC680 of 35 ps. A similar analysis was applied to the kinetics of the CB mutant of *Synechocystis* (Tian et al. 2013), where the rods consist of only the proximal PC hexamer. The three rise terms, assigned to PC, APC660 and PSI/PSII in this case, had lifetimes of 7, 25 and 77 ps. Although the slowest lifetime in this model is dominated by the coupling of APC and the photosystems, the rate-limiting step must still be equilibration within the PBS because practically the same lifetimes and DAES were observed in isolated PBS (Tian et al. 2012). This has been taken into account in the recent extended models of the kinetics of isolated PBS (van Stokkum et al. 2018) and PSI-deficient *Synechocystis* (Acuña et al. 2018a), wherein equilibration between cylinders in the PBS takes up to 200 ps and the time constant of EET from the terminal emitters in the basal APC cylinders to Chls in PSII is 20 ps. The model is fundamentally different from the earlier analysis of Mullineaux and Holzwarth (1991), who interpret the 180–220 ps decay in *Synechococcus* 6301 cells as reflecting slow EET between APC and PSII, where charge separation occurs on a timescale of about 40 ps. However, the authors assumed that the emission originated from the terminal emitters, which is incompatible with the 660 nm emission maximum of the 184 ps DAES.

The longer-lived component (400–500 ps) can safely be attributed to electron transfer in PSII, owing to its maximum at 680 nm. However, also in this case, the observed secondary peaks in the 640–660 nm region indicate that even this component is not associated with PSII only but also reflects decay of excitations in the PBS (Supplementary Fig. S5). We therefore propose that the 600 ps fluorescence decay lifetime in isolated PBS represents an intrinsic component of the antenna kinetics that is also observed in vivo. In agreement with this, we notice that the amplitude of the 500 ps component in cells is diminished at 640–660 nm relative to 680 nm upon predominant excitation of Chls at 460 nm (Fig. 5, Supplementary Fig. S5). On the other hand, the amplitude at 680 nm increases upon Chl excitation relative to the 200 ps component (APC), corroborating that the emission at this wavelength originates from Chls.

As discussed above, there are three different interpretations in the literature regarding the main APC decay time of 180 ps reported here for *Anabaena* filaments: equilibration in the PBS, EET from APC to PSII and trapping in PSII. These three scenarios can be referred to as migration-limited, transfer-to-trap-limited and trap-limited, respectively. We can argue against a pure trap-limited scenario, because trapping in the isolated PSII core occurs with a main lifetime of 30–50 ps (Holzwarth et al. 2006), i.e. significantly faster than the observed decay upon PBS excitation. The lifetime must then reflect energy equilibration in the PBS–PSII supercomplex. Furthermore, if we assume rapid and nearly irreversible EET from APC to the PSII antenna, then APC should be depopulated faster, whereas the data show slowly decaying emission at 660 nm. On the same grounds we can exclude the transfer-to-trap-limited case where the single rate-limiting step is coupling of the terminal emitters (APC680) to Chls in PSII or PSI—the bulk APC660 would be depopulated faster than APC680. In principle, the fluorescence kinetics of isolated PBS and of intact cells with open PSII RCs could be interpreted in the frame of a model where APC660 and APC680 equilibrate rapidly and the rate-limiting step is between APC and Chls (Mullineaux and Holzwarth 1991).

### Variable PSII fluorescence

The contribution of PSII charge separation to the overall excitation dynamics in cells can be inferred by comparing the fluorescence kinetics recorded with open and closed PSII RCs (Mullineaux and Holzwarth 1993, 1991; Remelli and Santabarbara 2018; Santabarbara et al. 2019). Our results well reproduced what has been reported previously for *Synechocystis*—in “*F*_m_” conditions, the short-lived decay components mainly associated with the PBS were barely affected, the main decay component lifetime (160–190 ps) remained virtually the same, whereas the ~ 400 ps lifetime increased along with its relative amplitude. The invariance of the 160–190 ps lifetime rules out purely trap-limited dynamics. However, in our experiments the shape of the 180–190 ps DAES varies—in *F*_m_ conditions, its amplitude at 680 nm is higher, suggesting decay of PSII antenna states and not only APC. This leads to a model where APC680–Chl transfer is fast and the kinetics is determined by a complex energy migration in the PBS (van Stokkum et al. 2018). Then again, it is surprising that the overall kinetics is virtually the same in species like *Anabaena* and *Synechocystis* having different organizations of the PBS and varying in the number and size of PBPs in the rods and the core. Ultimately, target analysis based on simultaneous model fitting of the isolated complexes and intact filaments at different excitation conditions and in different states of PSII should help distinguish between these scenarios.

Interestingly, the longest-lived fluorescence component (1.5–1.7 ns) increases its contribution under *F*_m_ conditions (with DCMU and additional background illumination). While the long-lived emission at 680 nm has been earlier observed (Remelli and Santabarbara 2018) and attributed to the closed PSII RCs, we also found increased relative amplitudes at 640 nm, i.e. from PC (Supplementary Fig. S6). In *F*_o_ conditions, this component originates from around 3% of uncoupled PC rods, but the number increased to 10% when the sample was continuously illuminated. Because of the long lifetime, the component has the dominant contribution to the stationary fluorescence at 640–660 nm in *F*_m_ conditions and, consequently, to the variable fluorescence spectrum and the *F*_v_/*F*_m_ spectrum (Supplementary Fig. S7). The variable fluorescence presumably originates only from PSII and its associated antenna. If this is true, it would follow that the emission at 650 nm is a result of energy equilibration between PSII and the PBS. However, the excitation dependence of the variable fluorescence is an evidence against such an interpretation. As pointed out by Remelli and Santabarbara (2018), we can expect the *F*_v_ spectrum to be independent from excitation wavelength, if there is rapid equilibration between the two systems (PBS and PSII). This has indeed shown to be the case in different cyanobacterial species (Remelli and Santabarbara 2018; Santabarbara et al. 2019). In contrast, in our experiments the 640 nm peak in the *F*_v_ spectrum is observed upon preferential PBS excitation at 580 nm (Supplementary Fig. S7). Moreover, slow back transfer to the PBS would incur a rise term at 640 nm upon Chls excitation, which is not observed. Therefore, it seems more likely that the cause of this emission is PBS or PBS rods that are uncoupled from PSII in *F*_m_ conditions. This may be a consequence of the measurement protocol using a combination of DCMU and prolonged background illumination and related to the previously reported light-induced detachment of PBS (Chukhutsina et al. 2015; Tamary et al. 2012; Stoitchkova et al. 2007). This phenomenon will be investigated further.

## Conclusions

The kinetics of EET in the PBS of *Anabaena* PC 7120 was found to be remarkably similar to other cyanobacterial PBS, notably that of *Synechocystis*. This is somewhat surprising considering the differences in composition and architecture of the two systems. The effective rod–core equilibration time was found to be about 125 ps and equilibration with the terminal emitters in APC was faster than the experiment time resolution.

The picosecond excited state dynamics of isolated tetrameric PSI from *Anabaena* can be described with two lifetimes of about 10 ps and 40 ps that represent energy equilibration with low-energy “red” Chl forms and trapping of the equilibrated excitations, respectively. The slower trapping compared to other cyanobacterial species might be related to the higher number and lower-energy of the “red” Chls in *Anabaena*. Because the number of Chls associated with PSI exceeds fivefold the Chls in PSII, the fluorescence kinetics of intact cells is dominated by PSI, allowing us to resolve both the equilibration with the red Chls and trapping kinetics in PSI in vivo.

In intact filaments, virtually all energy absorbed by the PBS was rapidly transferred to the photosystems. We found about 3% of PC uncoupled from the rest of the system, which, however contributed to 22% of the fluorescence emission at 650 nm. No uncoupled PEC was detected. EET within the PBS is well resolved in the intact filaments and trapping of PBS excitations occurs on a timescale of 170–200 ps, similar to previous reports. The lifetime is most likely determined by EET within the PBS (primarily the APC core) and between the PBS and the membrane complexes. The inverted kinetics regime, where EET to the photosystems is effectively slower than trapping by charge separation, makes it very difficult to directly resolve the two photosystems upon PBS excitation, as the Chl excited states are depopulated faster via charge separation than they are populated by the PBS. In principle, simultaneous model fitting of the results obtained with selective excitation of PBS and Chls could resolve this ambiguity; however, this is by far not trivial, as one must consider the coexistence of an a priori unknown number of PSI and PSII that have or have no contact with PBS (van Stokkum et al. 2018). Kinetic modelling of this kind, performed with the data reported here, will be presented in a subsequent work.

## Electronic supplementary material

Below is the link to the electronic supplementary material.

Supplementary file1 (DOCX 1593 kb) Supplementary figures: SDS-PAGE of isolated PSI, representative fluorescence decays of intact cells, normalized DAES of intact cells with 580 nm excitation, comparison of DAES of intact cells with open and close PSII RCs, preferentially exciting PBS or Chls, reconstructed variable fluorescence spectra, time-resolved emission spectra of PSI.

## Data Availability

The time-resolved fluorescence data reported here are deposited at Mendeley Data (Akhtar and Lambrev 2019).
