# Supplementary material for: Time-resolved fluorescence study of excitation energy transfer in the cyanobacterium Anabaena PCC 7120
Source: Photosynth Res. 2020 Feb 19;144(2):247–59. doi: 10.1007/s11120-020-00719-w (PMC7203587; doi:10.1007/s11120-020-00719-w)
Supplement: Supplementary file 1 — Supplementary file1 (DOCX 1593 kb) Supplementary figures: SDS-PAGE of isolated PSI, representative fluorescence decays of intact cells, normalized DAES of intact cells with 580 nm excitation, comparison of DAES of intact cells with open and close PSII RCs, preferentially exciting PBS or Chls, reconstructed variable fluorescence spectra, time-resolved emission spectra of PSI. [file 11120_2020_719_MOESM1_ESM.docx]

Photosynthesis Research

Time-resolved fluorescence study of excitation energy transfer in the cyanobacterium *Anabaena* PCC 7120

Parveen Akhtar, Avratanu Biswas, Nia Petrova, Tomas Zakar, Ivo van Stokkum, Petar H. Lambrev^*^

^*^ Biological Research Centre, Szeged, E-mail [lambrev.petar@brc.hu](mailto:lambrev.petar@brc.hu)

# Supplementary Figures


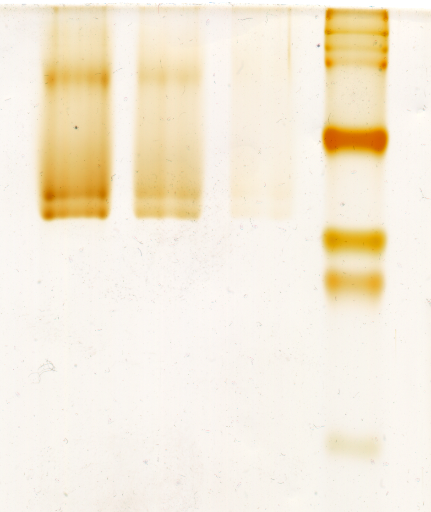


100%

70%

40%

ProSieve™

70 kDa

40 kDa

140 kDa

PsaA/B

**Fig. S1** SDS-PAGE of isolated Photosystem I tetramers. The gel was prepared with 12.5% acrylamide:bis-acrylamide (30:1) and polymerized with 0.05% ammonium persulfate and 0.0.05% N,N,N′,N′-Tetramethylethylenediamine (TEMED). The stacking gel contained 0.5 M Tris-HCl (pH 6.8) and 0.4 % SDS and in separation gel was 1.5 M Tris-HCl (pH 8.8) and 0.4 % SDS. The electrode buffer (pH 8·3) contained 0.025 M Tris and 0.192 M glycine and 0-1 % SDS. The running buffer contained 250 mM Tris, 1.92 M Glycine 1% SDS. Before loading the samples were mixed with 0.2 M Tris-HCl (pH 6·8), 2 percent SDS, 10 per cent glycine, 5 perceut β-mercaptoethanol and 0.001 % bromophenol blue as the dye. The proteins were completely dissociated by incubating the sample at 60 °C for 10 min. Electrophoresis was started with a current of 5-15 mA until the bromophenol blue marker started to move and then increased to 25-50 mA. The proteins were fixed with 40% methanol and 0.1% Formaldehyde overnight, stained for 10 h at 37 °C with 0.1 % (w/v) silver nitrate. The gel was destained by repeated washing with water. The assignment of the proteins was based on the molecular weights and is similar to Kato et al. (Nat. Commun. 10:1-9, 2019)

**Fig. S 2** Room temperature fluorescence emission spectra of isolated PBSs and PSI complexes. (a) isolated PBSs, excitation wavelength 550 nm; (b) isolated tetrameric PSI, excitation wavelength 460 nm.


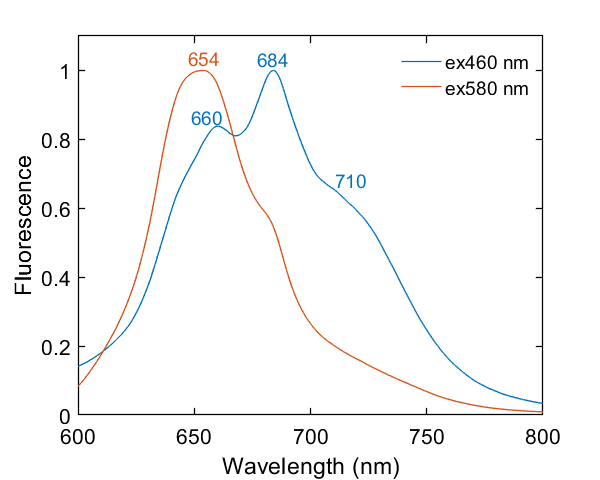


**Fig. S 3** Room temperature fluorescence emission spectra of intact *Anabaena* PCC 7120 filaments.

**Fig. S4** Fluorescence kinetics of intact *Anabaena* PCC 7120 filaments*.* (a) Fluorescence decay trace at 660 nm emission (black) with 580 nm excitation, (b) Fluorescence decay trace at 720 nm emission (black) with 460 nm excitation. The five-exponential fit (blue), IRF (red) and weighted residuals (lower subplot)

**Fig. S5** DAES of *Anabaena* filaments, 580 nm excitation (data from Figure 4a), normalized to their respective maximum.

**Fig. S6** DAES of intact *Anabaena* filaments with open (F_o_) or closed (F_m_) PSII RCs, upon excitation at either 460 or 580 nm. The two shortest-lived decay components are omitted from each dataset. The spectra are normalized to unity fluorescence intensity at the emission maximum.

**Fig. S7** Variable fluorescence spectra of *Anabaena* filaments upon 460 or 580 nm excitation. Upper panels: stationary emission spectra reconstructed from the DAES obtained from cells with open (F_o_) or closed (F_m_) PSII RCs and the variable fluorescence spectra F_v_ = F_m_ – F_o_. Lower panels – normalized variable fluorescence spectra F_v_/F_m_

**Fig. S8** Time-resolved emission spectra of PSI tetramers at selected time points recorded by time-correlated single photon counting at room temperature. The fluorescence kinetics at different emission wavelengths are identical below 670 nm, while the maximum shifts from 688 nm to 720 nm after 40 ps.
